# Interaction of lncRNAs with mTOR in colorectal cancer: a systematic review

**DOI:** 10.1186/s12885-023-11008-9

**Published:** 2023-06-06

**Authors:** Marziyeh Sadat Moslehian, Roya Shabkhizan, Mohammad Reza Asadi, Ahad Bazmani, Mahdi Mahdipour, Sanya Haiaty, Reza Rahbarghazi, Ebrahim Sakhinia

**Affiliations:** 1 Infectious and Tropical Diseases Research Center, Tabriz University of Medical Sciences, Tabriz, Iran; 2 Department of Medical Genetics, Faculty of Medicine, Tabriz University of Medical Sciences, Tabriz, Iran; 3 Department of Pathobiology, Faculty of Veterinary Medicine, Ferdowsi University Of Mashhad, Mashhad, Iran; 4 Stem Cell Research Center, Tabriz University of Medical Sciences, Imam Reza St., Golgasht St, Tabriz, Iran; 5 Department of Biochemistry and Clinical Laboratories, Tabriz University of Medical Sciences, Tabriz, Iran; 6 Department of Applied Cell Sciences, Faculty of Advanced Medical Sciences, Tabriz University of Medical Sciences, Tabriz, Iran; 7 Tabriz Genetic Analysis Centre (TGAC), Tabriz University of Medical Sciences, Tabriz, Iran

**Keywords:** Colorectal cancer, LncRNAs, MTOR Signaling Pathway, Systematic review

## Abstract

Colorectal cancer (CRC) is the third most widespread cancer and the fourth leading lethal disease among different societies. It is thought that CRC accounts for about 10% of all newly diagnosed cancer cases with high-rate mortality. lncRNAs, belonging to non-coding RNAs, are involved in varied cell bioactivities. Emerging data have confirmed a significant alteration in lncRNA transcription under anaplastic conditions. This systematic review aimed to assess the possible influence of abnormal mTOR-associated lncRNAs in the tumorigenesis of colorectal tissue. In this study, the PRISMA guideline was utilized based on the systematic investigation of published articles from seven databases. Of the 200 entries, 24 articles met inclusion criteria and were used for subsequent analyses. Of note, 23 lncRNAs were prioritized in association with the mTOR signaling pathway with up-regulation (79.16%) and down-regulation (20.84%) trends. Based on the obtained data, mTOR can be stimulated or inhibited during CRC by the alteration of several lncRNAs. Determining the dynamic activity of mTOR and relevant signaling pathways via lncRNAs can help us progress novel molecular therapeutics and medications.

## Introduction

According to the released statistics in 2019, colorectal cancer (CRC) is the third foremost pervasive malignancy in cancer patients. It is projected that CRC causes over 1.8 million newly diagnosed cases with an approximate annual death of 900,000 [1, 2]. Due to recent progress in cancer therapy, the survival rate for CRC patients has been dramatically improved. Despite these advances, the therapeutic outcome under progressive CRC conditions is suboptimal based on a five-year survival rate in 12% of CRC cases [3–5]. Molecular investigations have indicated that the incidence and development of CRC is an intricate process with the involvement of exogenous and endogenous variables [6, 7]. For instance, recent investigations in molecular pathological epidemiology revealed a close association between dietary and lifestyle factors with the risk of CRC. It is suggested that smoking, alcohol drinking, processed meat, genetic predisposition, and some therapeutic agents such as aspirin can increase the chance of CRC in human [8, 9]. Hypermutation has been detected by large-scale sequencing in CRC samples, especially in association with substantial microsatellite instability (MSI) caused by hypermethylation and suppression of the *MLH1* gene [10]. Likewise, *APC*, *TP53*, *SMAD4*, *PIK3CA*, and *KRAS* are the candidate genes most often mutated [11]. To date, numerous attempts have been done to recognize the molecular processes and signaling pathways implicated in CRC development and progression [12–15].

The mammalian (or mechanistic) target of rapamycin (mTOR) is a critical constituent of a signaling pathway that controls varied cell activities such as progression and proliferation, metabolism, motility, phenotype acquisition, and angiogenesis [16, 17]. mTOR is a member of the phosphoinositide 3-kinase-related kinases family, which has a substantial impact on CRC [18, 19]. Regarding the central role of mTOR in CRC pathophysiology, future studies should focus on the elucidation of mTOR activity in CRC cases.

Several studies have shown that several long non-coding RNAs (lncRNAs) have a role in the regulation of the mTOR signaling pathway [20]. lncRNAs are transcripts that include more than 200 nucleotides and lack protein-coding capabilities [21]. Some of the lncRNAs may be transcribed by RNA polymerase II with comparable features to messenger RNAs (mRNAs) [22]. Transcribed or spliced RNAs from lncRNAs can alter the activity of several genes at multiple levels such as transcription, translation, and protein modification [23]. Following the progression of CRC, oncogenic lncRNAs can stimulate tumor activity, while other lncRNAs with a role as tumor suppressors inhibit tumor activity [24]. Data suggested that overexpression of certain lncRNAs is allied with poor prognosis and metastatic behavior in CRC patients [25]. To this end, therapies targeting lncRNAs may be potential approaches in CRC patients [26]. RNA sequencing data from the TCGA dataset have indicated that about 200 lncRNAs expressed differentially in CRC patients [27]. In particular, mTOR is a potential target that is affected by lncRNAs in CRC cases. Whether and how lncRNAs influence mTOR has been the subject of the area. In this systematic review, mTOR-associated lncRNAs were highlighted using literature database potentials to find possible correlations between lncRNA expression and mTOR regulation in CRC cases.

## Methods and materials

### Search strategy

This systematic review was carried out under the PRISMA principles [28]. The relevant electronic databases were comprehensively searched for all published research until October 25, 2022. Using the first search’s keywords, MeSH or Emtree terms, PubMed, Embase, Scopus, Web of Science, and Cochrane were searched. Google Scholar and ProQuest were searched for grey literature and unpublished studies. “Colorectal neoplasm*” and “Colorectal cancer” were essential search terms in a PubMed systematic search for CRC.

### Study selection and assessment of studies

Following the database search, all discovered studies were imported into Endnote Version 20.2.1, and duplicates were omitted. According to inclusion criteria, the titles and abstracts of the remaining publications were assessed, and all studies containing mTOR-associated lncRNAs in CRC were included in the analysis. The included papers met the following criteria as follows; the original research describing lncRNAs and their interaction with mTOR in CRC was published in English. Exclusion criteria included non-CRC or other cancer types. Studies that did not use human specimens, cell lines, or animal models, and lacked data to verify the mTOR-associated lncRNAs using a molecular technique were also excluded.

### Data extraction

The essential data was extracted from the study using a self-administered data extraction approach. The authors, year, country, study design, lncRNAs, dysregulation, mTOR regulation, and major findings were mentioned.

## Results

### Search results

**Fig. 1:**
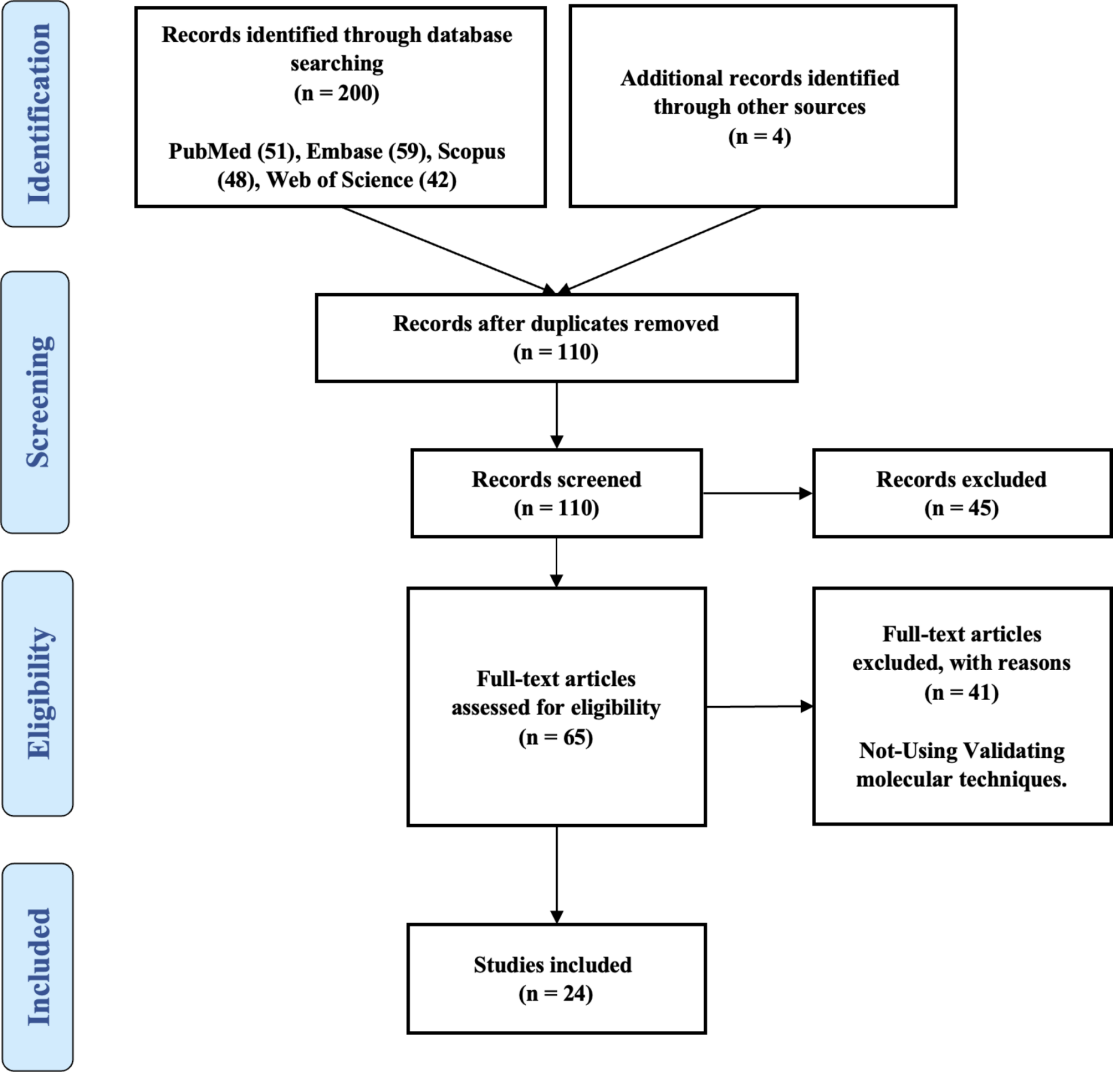
Search strategy flow chart based on the PRISMA flow diagram

Figure 1 represents a flowchart depicting the processes required to locate qualifying research. A total of 200 items from numerous sources were collected, of which 94 were duplicates. Eighty-two studies were eliminated due to the lack of significant data in the field. After the completion of the review, the remaining 24 publications were subjected to assessment purposes and the findings are shown in Table 1. The format of the conducted studies, types of lncRNAs affecting mTOR, their dysregulation, and direct and indirect impact on mTOR are classified in detail.

**Table 1:** mTOR-associated lncRNAs in CRC.

Study design	LncRNAs	Dysregulation	mTOR regulation	Major findings	Ref
Case-control Cell culture Animal model	*RP11-708H21.4*	Down-regulation	Direct inactivation	Increased expression of *RP11-708H21.4* reduces cell motility and invasion, induces cell death, and increases CRC cell sensitivity to 5-FU. Increased *RP11-708H21.4* expression inhibited the AKT/mTOR pathway and inhibited CRC xenograft tumor development in vivo.	[121]
Case-control Cell culture Animal model	*ZFAS1*	Up-regulation	Indirect activation	*ZFAS1* acted as a sponge to enhance the expression of *VEGFA* by binding directly to *miR-150-5p*, which is important in the activation of EMT and the AKT/mTOR signaling pathway.	[95]
Case-control Cell culture	*PVT1*	Up-regulation	Direct activation	*PVT1* overexpression may play a critical role in CRC multi-drug resistance by increasing the mRNA and protein expression levels of *MRP1*, P-glycoprotein, mTOR, and Bcl2.	[106]
Case-control Cell culture	*SNHG7*	Up-regulation	Indirect activation	*SNHG7* and *GALNT7* compete for *miR-34a* binding to activate the PI3K/Akt/mTOR pathway. As a ceRNA, *SNHG7* sponged *miR-34a* in CRC cell lines to regulate *GALNT7* expression, hence facilitating cell proliferation and metastasis.	[98]
Case-control Cell culture	*LncRNA-422*	Down-regulation	Direct inactivation	*lncRNA-422* functions as an anti-oncogene in CRC cells, decreasing cell growth, migration, and invasion while enhancing cell death, and its actions may be mediated by the PI3K/AKT/mTOR signaling pathway.	[122]
Case-control Cell culture Animal model	*BFAL1*	Up-regulation	Indirect activation	By competitively sponging *miR-155-5p* and *miR-200a-3p*, *BFAL1* modulates *RHEB* expression and regulates the RHEB/mTOR signaling pathway.	[99]
Case-control Cell culture Animal model	*TTN-AS1*	Up-regulation	Indirect activation	*TTN-AS1* was able to interact directly with *miR-497*, and co-transfection with *miR-497* mimics inhibited PI3K/Akt/mTOR signaling.	[100]
Cell culture	*UCA1*	Up-regulation	Direct activation	Cancer-associated fibroblasts induced *UCA1*, which controls the downstream critical effectors of cell growth and metastasis in CRC cells in collaboration with mTOR	[110]
Case-control Cell culture	*SNHG6*	Down-regulation	Direct inactivation	By targeting *ETS1*, *SNHG6* suppressed cell growth and metastasis and established preliminary interactions with the PI3K/AKT/mTOR signaling pathways	[123]
Case-control Cell culture Animal model	*HOTAIR*	Up-regulation	Indirect activation	The *HOTAIR/miR-326/FUT6* axis mediates CRC progression through 1, 3-fucosylated *CD44* and the PI3K/AKT/mTOR pathway.	[101]
Cell culture	*UCA1*	Up-regulation	Direct inactivation	Downregulation of *UCA1* inhibited autophagy, decreasing cell growth and increasing apoptosis. *UCA1* knockdown inhibited autophagy in 293T and Caco2 cells while activating the AKT/mTOR pathway.	[125]
Cell culture	*NBR2*	Down-regulation	Indirect inactivation	*NBR2* levels rose considerably in curcumin-treated CRC cells. *NBR2* knockdown reduced the stimulation of adenosine monophosphate-activated protein kinase and inhibition of the mTOR signaling pathway produced by curcumin.	[124]
Case-control Cell culture Animal model	*TINCR*	Up-regulation	Indirect activation	*TINCR*, functioning as a sponge for *miR-7-5p*, may enhance the advancement of CRC through a *miR-7-5p*-mediated PI3K/Akt/mTOR signaling pathway.	[102]
Case-control Cell culture Animal model	*DLX6-AS1*	Up-regulation	Direct activation	*DLX6-AS1* increased CRC cell growth, invasion, and migration while decreasing cell death via targeting the PI3K/AKT/mTOR signaling pathway.	[111]
Case-control Cell culture	*LINC00115*	Up-regulation	Direct activation	The *LINC00115/miR-489-3p* axis is associated with CRC cell growth, migration, and invasion via interacting favorably with the PI3K/Akt/mTOR signaling pathway.	[103]
Case-control Cell culture Animal model	*UCHL3*	Up-regulation	Direct activation	The PI3K/AKT/mTOR pathway is essential for *SOX12* expression to be mediated by *UCHL3*. Through the AKT/mTOR signaling pathway, *UCHL3* modulates *SOX12* and promotes tumor growth.	[93]
Case-control Cell culture	*UASR1*	Up-regulation	Direct activation	In CRC, the transcription factor *PAX5* encourages the expression of the lncRNA *UASR1*. Through the mTOR signaling pathway, highly elevated *UASR1* enhances the malignant growth of CRC.	[94]
Case-control Cell culture Animal model	*MALAT1*	Up-regulation	Indirect activation	Exosomal MALAT1 increased CRC cell malignancy by sponging *miR-26a/26b*, modulating *FUT4*, and activating the PI3K/Akt/mTOR pathway.	[104]
Cell culture	*CASC5*	Up-regulation	Direct activation	*CASC9* knockdown may induce mTOR-dependent autophagy by dramatically increasing AMPK phosphorylation and decreasing AKT and mTOR phosphorylation. The suppression of the AKT and mTOR signaling pathways may decrease cell proliferation and migration.	[112]
Cell culture	*RAMS11*	Up-regulation	Direct activation	*RAMS11* knockdown dramatically decreased CRC development through mTOR-dependent activation of autophagy.	[113]
Case-control Cell culture Animal model	*GAS5*	Down-regulation	Indirect activation	In CRC cells, the mTOR signaling pathway inhibited the production of *GAS5* and produced a negative regulatory feedback loop with *miR-34a*. The negative regulatory feedback loop *GAS5/miR-34a/SIRT1/mTOR* induced CRC cell macroautophagy.	[105]
Cell culture	*DLGAP1-AS2*	Up-regulation	Indirect activation	Accelerating radioresistance in rectal cancer cells, *DLGAP1-AS2* interacts with *E2F1* to increase *CD151* expression through the activation of the AKT/mTOR/cyclinD1 pathway.	[117]
Cell culture	*CRNDE*	Up-regulation	Direct activation	The reduction in p-mTOR protein expression demonstrates that *CRNDE* inhibition reduces the activity of AKT/mTORC1, as evidenced by the decrease in p-mTOR protein expression. The AKT/mTORC1 pathway may mediate the impact of *CRNDE* on the Warburg effect in HCT-116 cells.	[115]
Cell culture	*CKMT2-AS1*	Up-regulation	Direct inactivation	CRC cells were shown to have an elevated level of *CKMT2-AS1* expression. *CKMT2-AS1* knockdown lowers the survival of CRC cells through the AKT/mTOR signaling pathway.	[126]

### Study characteristics

**Fig. 2:**
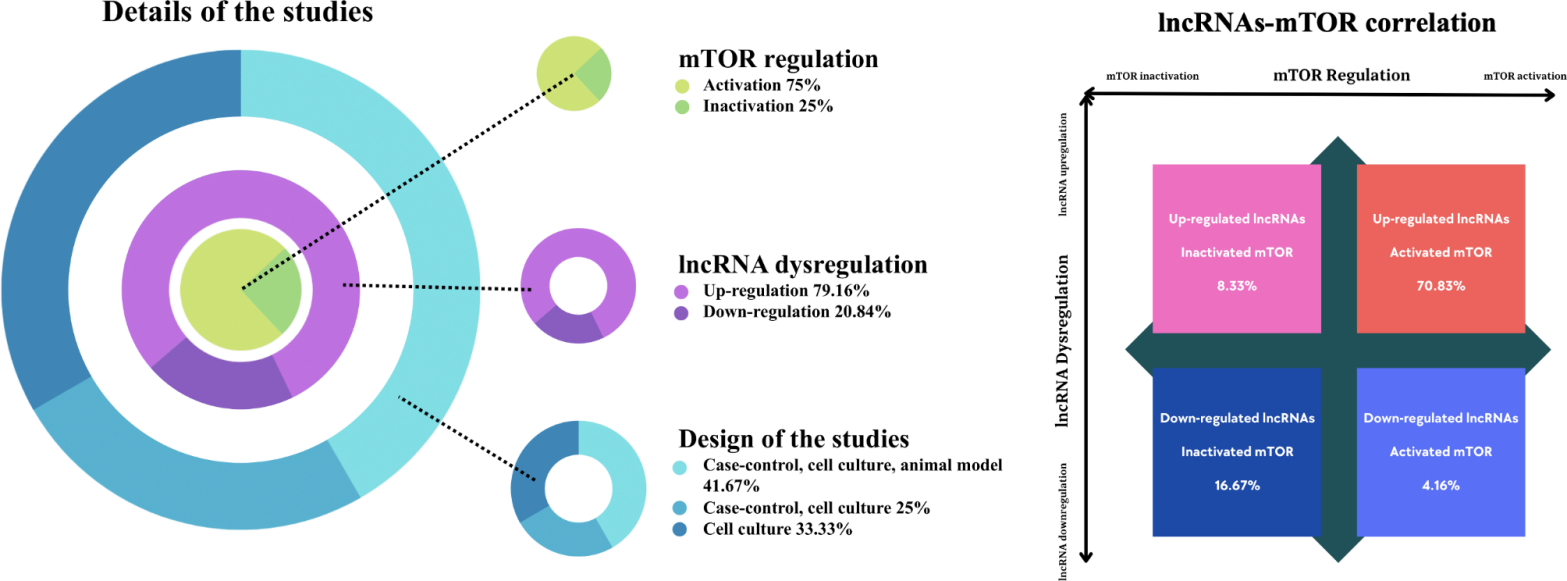
The charts of the study. Notably, mTOR was activated in 75% of studies under the influence of lncRNAs and inactivated in 25% of studies. Among these, 79.16% of studied lncRNAs are up-regulated, and 20.84% are down-regulated. The study design was also associated with 41.67% (case-control, cell culture, animal model), 33.33% (cell culture), and 25% (case-control, cell culture), respectively. Particularly, 70.83% and 8.33% of up-regulated lncRNAs were correlated with mTOR activation and inactivation, respectively. On the other hand, 16.67% and 4.16% of down-regulated lncRNAs were correlated with mTOR inactivation and activation, respectively

According to the obtained data, suitable publications were performed from 2018 to 2022. Figure 1 indicates the types of studies associated with human samples and cell line analysis and several model animals. Besides, our data indicated the close relationship between down- and up-regulated lncRNA with mTOR activity (Fig. 2).

## Discussion

### mTOR-associated carcinogenic pathway in CRC

Many upstream components of the mTOR signaling pathway, several oncogenes exert carcinogenic action through this axis. In the following, the most important pathways that are involved in CRC through mTOR will be discussed.

#### PI3K/AKT pathways

It was suggested that the insulin-like growth factor (*IGF*) signaling pathway mediates relevant biochemical reactions associated with nutrient sensing. The *PI3K* pathway promotes cell survival, and cell growth in response to signals from growth factor receptors like *EGFR*, *PDGFR*, and *IGF-1R* and the adhesion components including G-protein coupled receptors and integrins [29]. Class type I *PI3K* family members transform phosphatidylinositol 4, 5-bisphosphate or *PtdIns* (4, 5) P2 (*PIP2*) into phosphatidylinositol 3, 4, 5-trisphosphate or *PtdIns* (3, 4, 5) P3 (*PIP3*), therefore leading to the activation of *PDK1* and *mTORC2*. Phosphatase, tensin homolog, and *PTEN*, in particular, converse the mechanism by dephosphorylating *PIP3* to generate *PIP2*. *IGF-BP3* can bind to *IGF-1* and inhibits excessive *IGF1/AKT* signaling activation. It is considered that the phosphorylation of *AKT* residue at the Thr308 amino acid is performed by *PDK1*, Meanwhile, the phosphorylation of *AKT* residue on amino acid Ser473 is completed by *mTORC2*. *AKT* activity is completely activated by double phosphorylation [30].

In terms of cancer biology, the *PI3K/AKT* pathway is correlated with anaplastic changes via the regulation of cell proliferation, adhesion, transformation, and viability [31–33]. Mutations in the PI3K/PTEN/AKT pathway have been reported in CRC cell lines at a considerable percentage [34–37]. The *PIK3CA* mutation is seen in 15% of individuals with metastatic colorectal cancer (mCRC) [38]. The development of Cowden syndrome, which may lead to an increased lifetime risk of CRC, has been linked to germ-line *PTEN* ablation [39, 40]. Data show that CRC patients’ levels of *PI3K* subunits p85α and AKT1/2 were elevated, as well as mTORSer2448 and phosphorus-p70S6KThr389. The expression of p85α in phase IV tumors is likely to be significantly higher than in lower grades [34]. The GP130-mediated *mTORC1* activation process in mice was studied, and it was discovered that *JAK* and *PI3K* activity are required for *mTORC1* activation, which leads to colorectal tumorigenesis [41]. Inhibiting mTOR inhibits the phosphorylation of S6K and relieves RTK feedback repression, leading to *PI3K* and *AKT* activation [42, 43].

#### WNT pathway

The earliest recognizable precursor lesion in colorectal tissue is known as aberrant crypt foci (ACF) [44]. ACF originates from the epithelial cells of the intestine and intestine epithelium and can evolve into adenocarcinoma polyps and adenocarcinoma [45]. Typical functions of the tumor suppressor gene of Adenomatous polyposis Coli (APC) are to prevent the nuclear location of β-catenin and further degradation to suppress the canonical Wnt signaling pathway [46]. In human CRC, the signal axis of the β-catenin is strongly related to the control of *VEGF-A* expression. These features suggest a potential function for β-catenin in CRC angiogenesis [47]. In CRC cells, β-catenin has also been found to activate cyclin D1, leading to a neoplastic transformation [48]. APC mutations or depletion may result in fundamental stimulating of the Wnt signaling pathway which is thought of as an initial event in CRC. Based on molecular investigations, APC mutations have been linked to the development of more than one hundred adenomatous polyps [49–51]. The activation of Wnt is followed by the promotion of the TSC-mTOR axis [52]. It should be noted that the mTOR signaling pathway and the mTOR protein levels in Apc716-depleted mice increased. It has been observed that inhibiting the *mTORC1* pathway in APC mutant mice by administering the drug RAD001 (Everolimus) results in the reduction of intestinal polyps and death rate in animals [52, 53].

#### P53 pathway

The gene p53 is known as the protector of the genome. This gene mediates a wide range of stress responses, including DNA damage, stress related to energy and metabolism, hypoxia, oxidative stress, oncogenic stress, and ribosomal failure. p53 can control downstream components and perform tumor suppressor functions through cell cycle interruption, aging, DNA repair, and programmed cell death regulation [54]. Under typical conditions, p53 impedes the mTOR pathway in different ways. Based on the data, the dysregulation of the p53 pathway is the second critical step in CRC carcinogenesis which is characterized by the progression of adenoma to carcinoma [55, 56]. This feature may be initiated by mutations in the *TP53* gene or loss of the 17p chromosome [55, 56]. The *IGF-1/AKT* pathway, an upstream control mechanism of mTOR, is constantly monitored by the activity of p53 [57, 58]. By regulating *PTEN* transcription, p53 promotes *IGF-BP3* and suppresses mitogenic signaling [59, 60]. Furthermore, p53 generates Sestrin1/2 to inhibit mTOR by phosphorylating AMPK and *TSC2* in response to DNA damage and oxidation stress [61]. It has been indicated that p53 may inhibit mTOR activity in CRC cell lines directly by the regulation of AMPK-β1 and *TSC2*. In particular, *TSC2* mRNA levels increased due to the activation of p53 induced by -irradiation varies depending on cell types. Results demonstrated that p53 can induce *TSC2* in HCT116 cells and mouse colon tissue [62, 63]. Factor namely, *REDD1* is another p53 target gene with the potential to modulate the mTOR signaling pathway [64]. The activity of reactive oxygen species (ROS) and oxidative stress controls *REDD1*. It seems that *TSC1/2* activation caused by hypoxia requires *REDD1* [65].

### Potential lncRNAs in CRC

#### lncRNAs as oncogenes in CRC

Regarding the characteristics of lncRNAs, it is suggested that lncRNAs have the potential to serve as oncogenes [66, 67]. Cancer may be triggered by the stimulation of oncogenes and the silencing of tumor suppressor genes (TSGs) [68]. The structure of the tumor is composed of cells with dysregulated genes associated with growth and differentiation. Oncogenes have a decisive function in the stimulation of cell growth [69]. Oncogene changes may range from the appearance of new oncogenes to the upregulation of preexisting proto-oncogenes [70]. Due to the relative simplicity of detecting increased expression levels of lncRNAs and their functional importance in vitro and in vivo, more oncogenic lncRNAs have been found in CRC rather than tumor suppressor lncRNAs [71]. Notably, the following section will describe the most significant lncRNA oncogenic potential proven by practical investigations.

Through its interaction with *SMARCA1*, a vital component of the NURF chromatin remodeling complex, the oncogenic lncRNA *DLEU1* identified in CRC is necessary for the activation of *KPNA3* [72]. Several studies have indicated that the upregulation of *DLEU1* [72, 73] and *KPNA3* [72] in human CRC samples has been correlated with a poor prognosis. lncRNA *DLEU1* can inhibit CRC cell growth, motility, and invasion, indicating the relevance of the *DLEU1/SMARCA1/KPNA3* axis in the etiology of this cancer type [72]. Several investigations have confirmed the carcinogenic involvement of lncRNA *SLCO4A1-AS1* in CRC progression [74–76]. lncRNA *SLCO4A1-AS1* promotes cell progression, motility, invasion, and epithelial-mesenchymal transition (EMT) by regulating the Wnt/β-catenin and EGFR/MAPK signaling pathways [74, 76]. Besides, it has an oncogenic function by inducing autophagy through the *miR-508 3p/PARD3* axis [75]. According to independent studies, *CCAT1* is another carcinogenic lncRNA [77, 78]. This lncRNA induces EMT and has been linked to local invasion, tumor phase, and vascular growth [78]. Along with the above-mentioned lncRNAs, *NEAT1*, an oncogenic lncRNA, endorses CRC cell growth, motility, and invasion by binding to and altering the stability of the *DDX5* protein, leading to the activation of the Wnt/β-catenin signaling pathway [79]. The simultaneous overexpression of *NEAT1* and *DDX5* has been discovered to predict poor patient prognosis, making the *NEAT1/DDX5/Wnt/β-catenin* axis a potential remedial target in CRC [79, 80]. NEAT1 is the molecular sponge of the *miR-150-5p*, controlling the expression of *CPSF4* and modifying the sensitivity of CRC cells to 5-fluorouracil (5-FU) [80].

#### lncRNAs as tumor suppressors in CRC

It has been shown that the expression of many tumor suppressor lncRNAs is decreased or deleted in CRC samples and cell lines [81]. The suppression of these lncRNAs may result in the decrease of cell death rate and stimulates cell growth and proliferation [82]. *APC1* is a tumor suppressor of lncRNA, whose expression is stimulated by APC. *APC1* lncRNA downregulation has been found in advanced-stage CRC samples with further metastasis to lymph nodes, remote sites, and poor patient prognosis [83]. *APC* promotes the production of *APC1* lncRNA by blocking *PPARα* recruitment on its promoter. The overexpression of *APC1* lncRNA leads to the suppression of proliferation, motility, and angiogenesis in CRC cells via regulating exosome biogenesis and lowering *Rab5b* stability. Notably, exosome derived from *APC1*-knocked-down CRC cells promotes angiogenesis by inducing MAPK signaling [83]. In addition, the lncRNA *ST3Gal6-AS1*, which is produced from the sialyl transferase gene promoter region, is downregulated in CRC tissues relative to neighboring normal tissues [84]. It was shown that *ST3Gal6-AS1* enhanced the enrichment of *MLL1* in the sense gene promoter region, triggered the modification of H3K4me3, and promoted expression. The *ST3Gal6-AS1/ST3Gal6* axis promotes α-2, 3 sialylations and inhibits the PI3K/AKT pathway, producing Foxo1 nuclear localization in CRC cells [84]. According to in vitro analyses and animal studies, *ST3Gal6-AS1* has a function in suppressing CRC cell growth, motility, and the promotion of programmed cell death [84]. The found negative correlation between *ST3Gal6-AS1* lncRNA levels and CRC tumor volume, lymph node metastasis, distant metastasis, and tumor stage emphasizes the tumor suppressor function of *ST3Gal6-AS1* lncRNA [84].

Several different lncRNAs have tumor-suppressive roles in other malignancies. *MEG3* is well-known in this respect as a tumor suppressor lncRNA [85]. The decreased expression of *MEG3* in CRC samples, as in other malignancies, has been associated with enhanced cell growth and decreased apoptosis [86]. *LINC00152* is another lncRNA with contradictory outcomes across studies. Zhang et al. revealed lncRNA *LINC00152* decreased expression in CRC samples. They demonstrated the significance of this lncRNA in lowering cell viability and triggering cell death by influencing the *Ki-67*, *Bcl-2*, and *Fas* expression levels [87]. In a study, Wang et al. stated that *LINC00152* promotes CRC growth through interactions with *NCL* and *Sam68* [88]. Likewise, Bian et al. discovered that lncRNA *LINC00152* increases CRC cell growth and metastatic capability and causes resistance to 5-FU via inhibiting *miR-139-5p* [89]. *LINC00152* can control the expression of *FSCN1* by *microRNA-632* and *microRNA-185-3p* and promote the malignant properties of CRC cells [90].

### mTOR-associated lncRNAs involved in CRC

#### mTOR activation

mTOR pathway is recurrently activated in human cancers [91, 92]. The mTOR pathway governs some cellular progression, including proliferation, development, and metabolism via monitoring of the supply of metabolites and amino acids [16, 91, 92]. Most lncRNAs in CRC directly or indirectly activate mTOR to regulate cell potential in favor of malignancy. In this regard, Li et al. reported the overexpression of lncRNA *UCHL3* in CRC is directly correlated to the growth, migration, and invasion of CRC cells [93]. *UCHL3*-medicated tumor growth was significantly decreased by *SOX12* knockdown, indicating the function of *SOX12* in CRC. lncRNA *UCHL3* is the potential to activate the PI3K/AKT/mTOR pathway and regulate *SOX12* [93]. Likewise, Wang et al. discovered the overexpression of *UNC5B* antisense RNA 1 (*UASR1*) lncRNA under the regulation of *PAX5* [94]. The lncRNA *UASR1* mediates the malignant proliferation of CRC by activating mTOR and mTOR signal pathways [94].

**Fig. 3:**
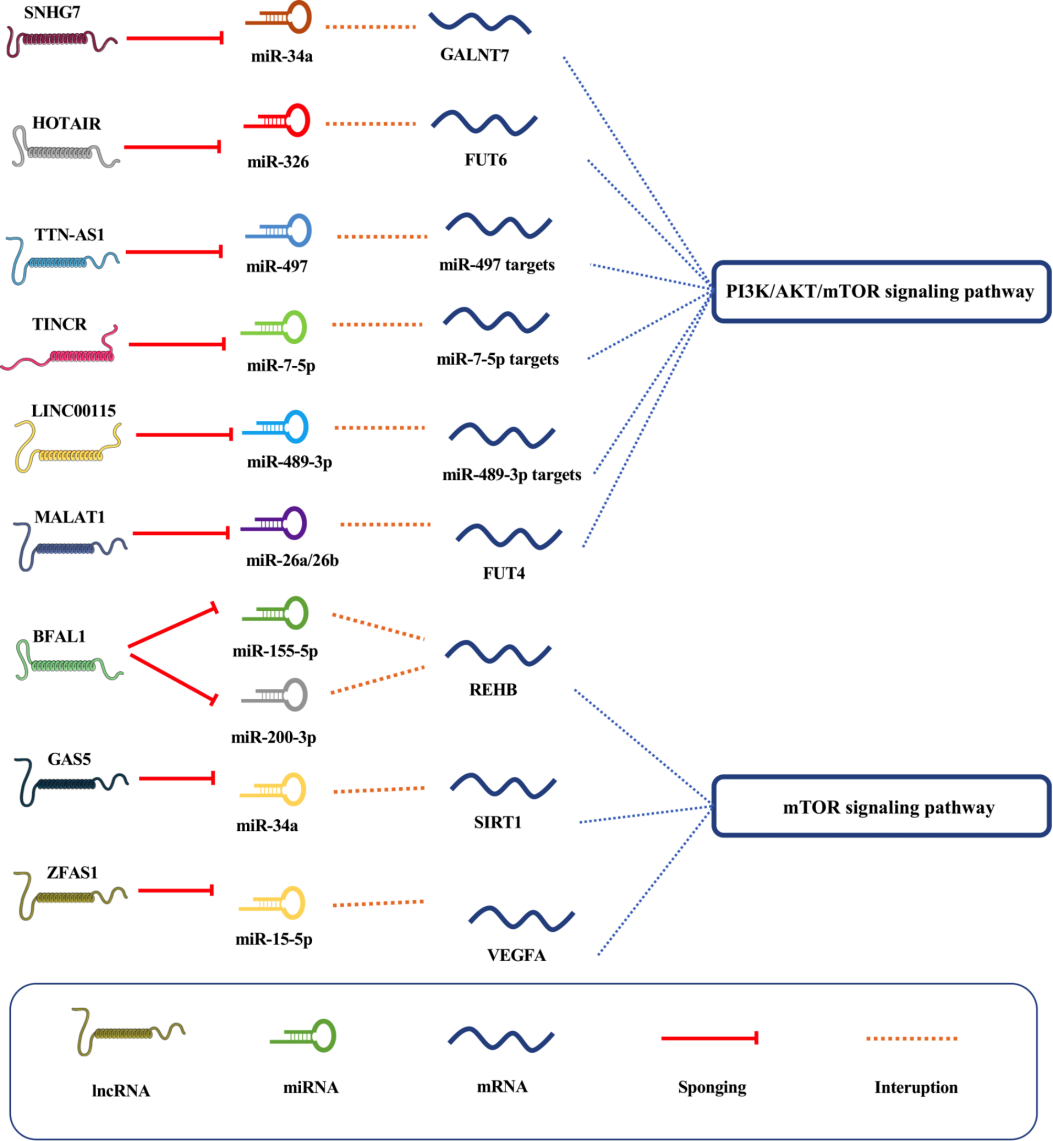
The schematic view of the lncRNA-associated ceRNA interaction influences mTOR-associated pathways in CRC. The lncRNAs act by inhibiting the target miRNAs to affect the target genes of these miRNAs. Interestingly, the effect of these dysregulated lncRNAs in the form of ceRNA axes on mTOR and mTOR-associated pathways has been measured in CRC.

Chen and colleagues indicated the expression of lncRNA *ZFAS1* in CRC cells and tissues coincided with *SP1* activation [95]. The silencing of lncRNA *ZFAS1* caused the inactivation of the AKT/mTOR signaling pathway and primarily hindered EMT in CRC cells [95]. It is postulated that the overexpression of lncRNA *ZFAS1* indicates its effectiveness as a competing endogenous RNA (ceRNA) on *miR-150-5p* [95]. In 2011, a research group suggested the name ceRNA for a novel method of interaction between RNAs [96]. It is proposed that a large-scale regulatory network covering the transcriptome would be derived from a sequence of complementary miRNAs called miRNA response elements (MREs) mediating communication concerning coding RNAs and non-coding RNAs [97]. According to the ceRNA hypothesis, if two RNA transcripts are controlled by a ceRNA-mediated process, their expression levels should be adversely associated with the levels of their respective target miRNAs and highly associated with each other [96]. Figure 3 provides a schematic view of the lncRNA-associated ceRNA axes in CRC with the effect on the mTOR and mTOR-associated pathways. Ten ceRNA axes have been proposed in the studies conducted on the mTOR signaling pathways through the mechanism of ceRNA with the participation of lncRNAs. In these axes, by sponging and inhibiting the function of the target miRNAs, lncRNAs prevent miRNAs’ effect on the target genes that are present in the mTOR-involved pathways, and by changing the mTOR pathway, they show their effect on the progression of CRC.

Li et al. introduced *SNHG7* as a ceRNA that competes for binding to *miR-34a* and activates PI3K/AKT/mTOR to facilitate CRC progression and metastasis [98]. Likewise, the overexpressed expression of *BFAL1* was discovered in CRC based on data from Bao et al. experiment [99]. *BFAL1* serves as a ceRNA to sponge *miR-155-5p* and *miR-200a-3p* and regulates the expression of *RHEB*, activates the RHEB/mTOR signaling pathway, and promotes tumor growth [99]. In this regard, Cui and colleagues discovered that the overexpression of *TTN-AS1* demonstrated its effect in enhancing CRC proliferation and invasion [100]. Notably, the upregulation of *TTN-AS1* increased PI3K/AKT/mTOR signaling in CRC cells through the regulation of *miR-497* [100]. It was suggested that *HOTAIR* inhibits *miR-326*, which in turn affects fucosyltransferase 6 (*FUT6*) [101]. The modulating interactions between *HOTAIR*, *miR-326*, and *FUT6* have been proven to activate PI3K/AKT/mTOR cascades and lead to the development of CRC [101]. In this regard, cell proliferation, invasion, and motility in CRC were all promoted due to the overexpression of *TINCR* following the activation of transcription factor *SP1* [102]. *TINCR* acts as ceRNA and sponges *miR-7-5p*, activating the AKT/mTOR signaling pathway in favor of malignant conditions in CRC [102]. Similarly, the up-regulation of lncRNA *LINC00115*, through influencing the PI3K/AKT/mTOR signaling pathway promotes tumor development, aggressiveness, and migration [103]. Feng et al. revealed that *LINC00115* targeted *miR-489-3p*, and down-regulation of *miR-489-3p* could release the biological effects [103]. Additionally, lncRNA metastasis-associated lung adenocarcinoma transcript 1 (*MALAT1*) directly binds to *miR-26a* and *miR-26b* and upregulates the enzyme fucosyltransferase 4 (*FUT4*) expression in CRC cell lines [104]. Thus, *MALAT1* enhances CRC formation via PI3K/AKT/mTOR pathway activation and *miR-26a/26b* sponging through *FUT4*-associated fucosylation [104]. Interestingly, Zhang et al. found the equilibrium condition of *GAS5*-mediated macroautophagy which may have had a protective impact on CRC cell death [105]. In CRC cells, the mTOR signaling pathway inhibited the production of *GAS5* and produced an adverse regulatory feedback axis with *miR-34a* [105].

In addition to the involvement of lncRNAs in ceRNA axes and influencing the activation of mTOR, lncRNAs can activate mTOR by regulating its expression or increasing the rate of mTOR phosphorylation. In this regard, Fan et al. revealed that the overexpression of plasmacytoma variant translocation 1 (*PVT1*) causes an increase in mTOR mRNA and protein levels [106]. Notably, the silencing of *PVT1* reversed the multi-drug resistance in CRC, considering that mTOR is one of the primary factors in cancer drug resistance [106]. Cancer-associated fibroblasts (CAFs) represent most of the cells in the microenvironment of the tumor and play a vital role in tumor progression, proliferation, spread, angiogenesis, and immunological function [107, 108]. CAFs are a diverse subset of fibroblasts triggered by tumor cells and are known to have particular biomarker functions that are considered predictive for cancer [109]. Jahangiri et al. revealed that CAFs acted as an inducing factor of urothelial carcinoma associated 1 (*UCA1*) in collaboration with mTOR governing the critical downstream inducers of CRC cell growth and metastasis [110]. Interestingly, Zhang et al. reported that the PI3K/AKT/mTOR signaling pathway could act a substantial role in the inhibition of cell apoptosis [111]. *DLX6-AS1* enhanced CRC cell growth, invasion, and motility but repressed cell-programmed cell death via activating the PI3K/AKT/mTOR signaling pathway [111]. Moreover, the overexpression of *CASC9* was reported in favor of promoting CRC cell carcinogenesis [112]. Islam Khan discovered that *CASC9* knockdown might increase mTOR-dependent autophagy by dramatically increasing AMPK phosphorylation while decreasing AKT and mTOR phosphorylation. The suppression of the AKT and mTOR signaling pathways may reduce cell growth and motility [112]. The potential of *RAMS11* in CRC advancement, migration, and invasion was also indicated [113]. *RAMS11* silencing can promote autophagy and apoptosis through AMPK phosphorylation and AKT and mTOR signaling suppression [113]. In HCT-116 cells, the role of mTOR in the Warburg effect was also established [114]. Yang et al. discovered that the overexpression of colorectal neoplasia differentially expressed lncRNA (*CRNDE*) in CRC cells are correlated with the modulation of programmed cell death, cell growth, and drug sensitivity [114]. In particular, silencing *CRNDE* in CRC cells reduced the Warburg effect, indicating that ATP synthesis, the level of lactic acid, glucose absorption, and expression of the related enzymes decreased [114]. Reduced mTOR phosphorylation leads to the suppression of *CRNDE* and inhibition of the AKT/mTORC1 pathway [114]. Utilizing the AKT and mTOR inhibitors in *CRNDE* overexpression-induced cells resulted in reducing ATP and lactic acid levels and glucose uptake, suppressing the Warburg effect [115].

Following radiation exposure, the stimulation of the PI3K/AKT/mTOR signaling pathway increases CRC cells’ resistance to radiation [116]. *DLGAP1-AS2* is overexpressed in CRC cells after being exposed to radiation in favor of radioresistance. Xiao and colleagues revealed that *DLGAP1-AS2*, indirectly motivates the AKT/mTOR/cyclinD1 signaling pathway, by regulating *E2F1* affecting *CD151* expression levels, and causing radiotherapy resistance in CRC cells [117].

#### mTOR inactivation

The contribution of activated mTOR and associated signaling pathways in the promotion of CRC in several aspects, including proliferation, migration, invasion, and resistance to treatment, is well-documented [118–120]. Remarkably, lncRNAs also served a critical role in inactivating mTOR and pathways involving mTOR in CRC. In this regard, it has been shown that the expression of the lncRNA *RP11-708H21.4* decreased abnormally in CRC, whose expression was closely related to the malignant clinical pathological features of CRC patients and poor prognosis [121]. Notably, the overexpression of lncRNA *RP11-708H21.4* could suppress CRC cell progression by stimulating G1 arrest and inhibiting the AKT/mTOR pathway [121]. Similarly, the results of the Shao et al. study stated the downregulation of anti-oncogene *lncRNA-422* in CRC samples [122]. *lncRNA-422* exerts its effects through the suppression of cell growth, motility, and invasion of CRC cells and the promotion of cell apoptosis by inhibiting the PI3K/AKT/mTOR signaling pathway [122]. Meng et al. signified prominent down-regulation of small nucleolar RNA host gene 6 (*SNHG6*) lncRNA in CRC [123]. Simultaneously, the proliferation rate was increased due to the overexpression of *ETS1*, one of the *SNHG6* targets. *SNHG6* suppressed the expression of *ETS1*, specifically targeting the 3′-UTR, and triggered an internal invasion path by downregulating the expression of PI3K/AKT/mTOR [123]. The neighbor of *BRCA1* lncRNA 2 (*NBR2*) was experiencing a decrease in CRC cells. However, Yu and colleagues revealed that curcumin raised the lncRNA *NBR2* expression level in response to treatment. In particular, *NBR2* decomposition eliminated the stimulation of protein kinases activated by adenosine monophosphate and the inhibition of the pathway of mTOR signals generated by curcumin [124].

It is noteworthy to mention that lncRNAs are also involved in mTOR inactivation. In this regard, Song et al. reported the upregulation of lncRNA *UCA1* in CRC cells, which is directly correlated with autophagy regulation [125]. The downregulation of lncRNA *UCA1* prompted autophagy repression and triggering of the AKT/mTOR signaling pathway, resulting in suppressing cell growth and promoting programmed cell death [125]. In another study, CAFs were identified as an *UCA1*-inducer in CRC cells; however, the overexpression of *UCA1* was associated with activating mTOR and was involved in promoting proliferation and metastasis [110]. Zhuang et al. highlighted the AKT/mTOR inactivation in response to the overexpression of *CKMT2-AS1* in CRC cells [126]. The silencing of *CKMT2-AS1* causes a reduction in CRC cell viability through the regulation of AKT/mTOR [126]. So far, the majority of studies in the field of the effects of pathways in which mTOR are involved by activating these pathways have been for the benefit of cancerous conditions in cancer. At the same time, looking at this pathway is more complicated, and studies of the effects of inactivating the pathways involved with mTOR have resulted. At this time, lncRNAs are only one of the critical factors that can affect mTOR, and it is this that makes the subject interesting for further studies. The current study faced some limitations. This review article was a preliminary attempt to address the possible relationship between the lncRNAs and mTOR status in CRC. First, studies on the influence of lncRNAs on mTOR and mTOR-involved pathways are in their infancy, and the reciprocal interaction between the mTOR and mTOR-involved pathways with lncRNAs needs further studies. Due to the lack of sufficient data in the field, a strong conclusion in terms of mTOR and lncRNAs should be done based on data acquired from more studies.

## Conclusion

According to their potential, lncRNAs can affect many biological pathways by the modulation of different signaling molecules. Several experiments have indicated the critical role of lncRNAs in the dynamic growth and expansion of cancer cells. Thus, these features make lncRNAs a suitable target for promoting the goals of cancer cells. In this regard, mTOR and mTOR-associated pathways are among the potential factors and pathways that are affected by lncRNAs in most cancers, especially CRC. In this current systematic review, we provided an inclusive overview of previous studies that use a validated molecular approach to assess the involvement of lncRNAs in mTOR and mTOR-associated pathways. Data indicated that mTOR and downstream cascade can be stimulated and/or inhibited via different types of lncRNAs in CRC, indicating the existence of an intricate interaction between the mTOR signaling pathway and lncRNAs. Due to the modulatory effects of lncRNAs in the dynamic growth of CRC via controlling mTOR and mTOR-associated pathways, it is highly recommended that the modulatory role of lncRNAs can be examined in other cancer types as well. The regulation of lncRNA transcription can be thought of as an effective modality in the control of specified cancer types such as CRC.

## Data Availability

All data are presented in this study.

## Abbreviations

5-FU: 5-fluororoacil
ACF: Aberrant crypt foci
APC: Adenomatous polyposis Coli
CRC: Colorectal cancer
EMT: Epithelial-mesenchymal transition
IGF: Insulin-like growth factor
lncRNAs: Long non-coding RNAs
mTOR: Mechanistic target of rapamycin
mRNAs: Messenger RNAs
MSI: Microsatellite instability
UASR1: UNC5B antisense RNA 1
